# Uncinate Process Length in Birds Scales with Resting Metabolic Rate

**DOI:** 10.1371/journal.pone.0005667

**Published:** 2009-05-27

**Authors:** Peter Tickle, Robert Nudds, Jonathan Codd

**Affiliations:** Faculty of Life Sciences, University of Manchester, Manchester, United Kingdom; Universidad Europea de Madrid, Spain

## Abstract

A fundamental function of the respiratory system is the supply of oxygen to meet metabolic demand. Morphological constraints on the supply of oxygen, such as the structure of the lung, have previously been studied in birds. Recent research has shown that uncinate processes (UP) are important respiratory structures in birds, facilitating inspiratory and expiratory movements of the ribs and sternum. Uncinate process length (UPL) is important for determining the mechanical advantage for these respiratory movements. Here we report on the relationship between UPL, body size, metabolic demand and locomotor specialisation in birds. UPL was found to scale isometrically with body mass. Process length is greatest in specialist diving birds, shortest in walking birds and intermediate length in all others relative to body size. Examination of the interaction between the length of the UP and metabolic demand indicated that, relative to body size, species with high metabolic rates have corresponding elongated UP. We propose that elongated UP confer an advantage on the supply of oxygen, perhaps by improving the mechanical advantage and reducing the energetic cost of movements of the ribs and sternum.

## Introduction

The avian respiratory system consists of a relatively rigid lung coupled with a series (in most birds) of nine compliant air-sacs [1]. The anatomical arrangement of these air-sacs facilitates the bellows-like movement of inspired air unidirectionally across the parenchymal tissue [2], [3]. Like all tetrapods birds face a possible mechanical constraint during simultaneous locomotion and ventilation [4], [5]. However, almost all extant birds and some non-avian maniraptoran dinosaurs exhibit osteological characters known as uncinate processes (UP). These bony projections on the vertebral ribs (oriented in the caudo-dorsal direction) play a key role in enabling simultaneous ventilation and locomotion. The function of the UP was thought to be linked with stiffening the rib cage [6], [7] or as a site for attachment of flight muscles [8]. However, their role as accessory breathing structures, first suggested by Zimmer [9], was confirmed in recent research [10]. The *Mm. appendicocostales* projects from the proximal edge of the uncinate process, and inserts onto the following vertebral rib [11]. Activity of the *Mm. appendicocostales* is associated with craniad movement of the ribs and a ventral displacement of the sternum during inspiration [10]. UP also provide a site for the attachment of projections from the *M. externus obliquus abdominus*, which pull the sternum dorsally during expiration [10]. Geometric modelling of the avian rib cage indicated that UP act as levers that improve the mechanical advantage for forward rotation of the dorsal ribs and therefore ventral movement of the sternum during inspiration [12]. Morphological variations in UP have been demonstrated to correspond to adaptations to different forms of locomotion [12]. Birds adapted to diving have the longest processes, flying and swimming birds have UP of intermediate length whilst the shortest UP occur in walking species [12]. Given the important respiratory function of the UP, variation in morphology suggests that differences in ventilatory mechanics may be driven by adaptation to locomotion. The presence of these processes in some non-avian maniraptoran dinosaurs has also been linked to avian-like breathing mechanics in these theropod ancestors of modern birds [13].

Respiration powers locomotion by providing metabolic energy. Resting metabolic rate (RMR) is often used to investigate the relationship between metabolism and body weight [14]. Lasiewski and Dawson [15] provided a review and re-analysis of published values of bird RMR. Using data from a range of species, RMR was reported to scale to the two-thirds power of body mass (RMR∝*M*_b_^2/3^), i.e. in proportion to body surface area [15], [16]. However, by re-analysing the scaling relationship in separate passerine and non-passerine groups, Lasiewski and Dawson [15] calculated that RMR scales as *M*_b_^0.72^. The projected 0.72 scaling coefficient found by Lasiewski and Dawson [15] is very similar to the ⅔ [17] or ¾ [16], [18] scaling component often used to describe the relationship between basal metabolic rate and body size for mammals. A common relationship between metabolism and body weight may therefore exist amongst endothermic vertebrates. Avian RMR has been reported to vary according to phylogeny, circadian rhythms and ecological variables [15], [19]–[23]. Furthermore, passerines have an elevated metabolic rate when compared to non-passerine species [15], while RMR is greater for birds during their period of normal activity [19], [20]. However, some controversy exists on the scaling relationship between passerine and non-passerine birds with studies either reporting [24] or not reporting [22], [25] a difference. The rate of energy metabolism in species adapted to life in aquatic environments is relatively high compared to arboreal birds, while nocturnal species have relatively low RMR [21]. Variation in energy metabolism within Aves suggests that adaptations in breathing mechanics may have occurred to meet the oxygen demands of varied lifestyles.

The supply of oxygen to gas exchange tissue is critically important for sustaining the metabolic rate of an animal. Numerous studies across a range of phyla have addressed the constraints upon oxygen delivery imposed by body size. For example, an examination of avian respiration by Lasiewski and Calder [26] highlighted the relative efficiency of the unique lung air-sac system.

UP are now confirmed to be important respiratory structures in birds involved in inspiration and expiration [10]. Here we disentangle the relationships between uncinate process length (UPL), *M*_b_ and RMR in birds adapted to different modes of primary locomotion. An understanding of the relationship between the UPL and metabolic rate will shed further light on the evolution of morphological variation in these processes.

## Materials and Methods

### (a) Specimens

Measurements of UPL were taken from the left-hand side of skeletons from 112 species using a Mayr digital calliper (16EX 150 mm, Product No: 4102400, Mayr GmbH, Germany). The length of process 4 was used in all statistical analyses. This process occurs in all specimens and has a relatively stable morphology [27]. Following the classification of Tickle et al [12], birds were grouped based upon their primary mode of locomotion and assigned to a single category; (1) walking (n = 13); birds which are flightless or incapable of sustained flight: (2) diving (n = 27); birds which are capable of diving underwater using foot or wing propulsion; and (3) non-specialist (n = 72); all other birds which fly and swim but are not flightless or capable of diving underwater. Body masses were taken from records of intact specimens or literature values [28].

### (b) Uncinate process length and body size

Reduced major axis (RMA) regression [29] was used to examine the relationship between UPL and *M*_b_. This procedure is appropriate for analysis of morphological characters because the variation in both x and y variables is taken into account [29], [30]. The slope and confidence intervals of the regression line were calculated as described by Sokal and Rohlf [29]. RMA equations for log_10_ transformed data were independently generated in each locomotor category. ANCOVA was used to test for significant differences between regression lines.

The potentially confounding effects of the birds having a shared phylogenetic ancestry were controlled by calculating independent contrasts [31]. The phylogeny of Livezey and Zusi [32] was used to generate contrasts for log_10_ transformed *M*_b_ and UPL data, with the CRUNCH facility in the Comparative Analysis of Independent Contrasts (CAIC) software, version 2.6.9 [33]. A punctuational model of evolution was assumed and therefore all branch lengths were set as equal. RMA regression equations were calculated [29] to explore the linear relationship between phylogenetically corrected UPL and *M*_b_. RMA regressions of independent contrasts were performed through the origin [33] for each locomotor mode.

### (c) Uncinate process length and metabolic rate

The relationship between RMR and UPL was studied in a subset of 35 species, taken from the previous dataset. These species were selected because corresponding RMR values are available [21], [34]. These species were again assigned to locomotor categories, walking, non-specialist and diving. Body masses and values of RMR (taken from [21], [34]) were log_10_ transformed, and only data corresponding to true RMRs, as defined by them, were used. To ensure that the regression equations describing the relationship between UPL and *M*_b_ in the species subset did not differ significantly from those in the larger dataset, RMA analyses were repeated. This procedure is important for validating the assumption that an association between process length and RMR can be extrapolated to the larger dataset. RMA regression was used to investigate the linear relationship between *M*_b_ and RMR. Finally, the scaling of RMR with UPL was explored using RMA regression.

General linear models (GLM) were used to assess the influence of locomotor mode, UPL and *M*_b_ on RMR. By accounting for the variation attributable to *M*_b_ and group structure, the GLM provides an estimate of the association between RMR and process length independent of *M*_b_. In the first analysis, locomotor category (diving, non-specialist and walking) was designated as a group factor, while *M*_b_ and UPL were the covariates (RMR = locomotor group+UPL+*M*_b_). A second GLM analysis repeated the prior method with the group factor comprising diving and non-specialist species. Lastly, the influence of UPL and *M*_b_ on RMR, in the absence of a grouping variable, was examined (RMR = UPL+*M*_b_).

A phylogenetically controlled analysis in the form of the GLM (above) was not possible. Therefore, to assess the influence of locomotor mode, UPL and *M*_b_ on RMR whilst controlling for phylogeny we employed the following method: first the linear relationship between log_10_ *M*_b_ and log_10_ UPL was determined (UPL = 0.2137 *M*_b_+0.4336). Residual UPL was then calculated by subtracting the length predicted from the log_10_ *M*_b_/log_10_ UPL relationship from actual UPL for each species. The relationship between log_10_ RMR and residual uncinate process length was then calculated for each of the three locomotor groups using CAIC. RMA regressions were calculated to assess the linear relationship between the independent contrasts generated for RMR and residual UPL within each locomotor mode.

GLM analyses were conducted in SPSS (SPSS v.15; SPSS Ltd, Chicago, IL, USA) and ANCOVAs, and RMA regressions in MATLAB® 2007b (The MathWorks, Inc., 3 Apple Hill Drive, Natick, MA). Ninety five percent confidence limits are displayed in parentheses immediately after the scaling exponent.

## Results

### Uncinate process length and body size

**Figure 1:**
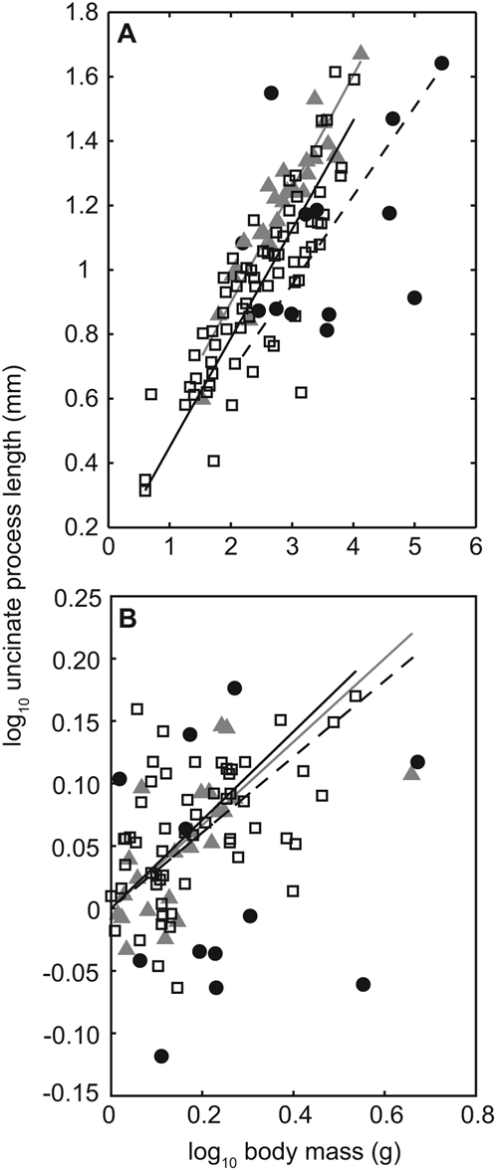
The relationship between uncinate process length (UPL) and body mass (a) species level analysis, b) comparative analysis using independent contrasts and the phylogeny of Livezey and Zusi [31]. Diving birds (grey solid triangles and grey regression lines), non-specialists (open squares and black regression lines) and walkers (solid circles and dashed regression lines). The equations describing the lines of best fit were *y* = 1.96 *x* ^0.35 (0.30−0.41)^ (*t* = 12.80, *n* = 27, *r* ^2^ = 0.85, *p*<0.001) and *y* = *x* ^0.33 (0.25−0.42)^ (*t* = 8.47, *n* = 23, *r* ^2^ = 0.68, *p*<0.001) for diving birds, *y* = 1.29 *x* ^0.34 (0.29−0.38)^ (*t* = 14.94, *n* = 72, *r* ^2^ = 0.71, *p*<0.001) and *y* = *x* ^0.35 (0.30−0.41)^ (*t* = 12.47, *n* = 57, *r* ^2^ = 0.63, *p*<0.001) for non-specialists, and *y* = 1.34 *x* ^0.28 (0.07−0.48)^ (*t* = 2.99, *n* = 13, *r* ^2^ = 0.15, *p*<0.02) and *y* = *x* ^0.30 (0.12−0.49)^ (*t* = 3.57, *n* = 12, *r* ^2^ = 0.06, *p*<0.005) for walkers in a & b respectively.

Analyses of 112 species indicated that, for all three locomotor modes investigated, uncinate process length scales isometrically with body mass (Figure 1a; Table 1), i.e. in all cases the regression line exponent did not differ significantly from 0.33. The regression line intercept differed between locomotor groups, indicating that processes are longest relative to *M*_b_ in diving birds, intermediate in non-specialists and shortest in walking birds (Figure 1a; Table 1) (ANCOVA: group, *F*_2,108_ = 14.16, *P*<0.001; *M*_b_, *F*_1,108_ = 169.54, *P*<0.001). Greater variation around the regression line was evident for the walking category, as indicated by the lower correlation coefficient, *r* (Figure 1a; Table 1). Scaling of UPL against *M*_b_ using independent contrasts suggested that when phylogeny is taken into account the effects were minimal and scaling was isometric (*M*_b_^0.33^) for all locomotor groups (Figure 1b; Table 1). Similar to the corresponding values estimated using the species level analysis above, variation around the regression line was greatest within the walking category (Table 1).

**Table 1:** Parameters described in the table are for scaling relationships of the form *y* = m *x* ^c^.

*y*	*x*	Analysis	Locomotor mode	*n*	*r*	m	RMA slope	95% CI
UPL	*M*_b_	Species	Walkers	13	0.38	1.34	0.28	0.07–0.48
			Non-specialists	72	0.84	1.29	0.34	0.29–0.38
			Divers	27	0.92	1.56	0.35	0.30–0.41
		Phylogenetically controlled	Walkers	12	0.24		0.30	0.12–0.49
			Non-specialists	57	0.80		0.35	0.30–0.41
			Divers	23	0.82		0.33	0.25–0.42
		Species (RMR subset)	Walkers	6	0.26	1.90	0.18‡	−0.13–0.49
			Non-specialists	21	0.91	1.20	0.38	0.30–0.46
			Divers	8	0.96	1.16	0.39	0.29–0.50
RMR			Walkers	6	0.98	0.64	0.68*	0.51–0.84
			Non-specialists	21	0.98	0.62	0.71*	0.63–0.79
			Divers	8	1.00	0.65	0.70*	0.66–0.75
	*U*_p_		Walkers	6	0.17	0.06	3.69‡	−2.92–10.30
			Non-specialists	21	0.89	0.43	1.53*	1.11–1.95
			Divers	8	0.96	0.50	1.78*	1.30–2.27

‡ Indicates the regression was not significant at p = 0.05 (i.e., the slope of the relationship did not differ from zero).

* Indicates the slope differs from that expected for isometric similarity.

Within the data subset of species where RMR values are also available (*N* = 35: diving = 8; non-specialist = 21; walking = 6) UPL scaled isometrically with *M*_b_ in the diving and non-specialist birds (Table 1). In contrast, the relationship between UPL and *M*_b_ in the walking group (Table 1) was not significant. Nonetheless, the overall trend is similar to that in the full data set.

While a significant positive relationship was found between RMR and *M*_b_, this relationship did not differ between groups (ANCOVA: group, *F*_2,29_ = 1.19, *P* = 0.317; *M*_b_, *F*_1,29_ = 762.18, *P*<0.001; group*M_b_, *F*_2,29_ = 0.13, *P* = 0.882). The group and *M*_b_ interaction term was included to ensure that the slope of RMR against *M*_b_ for the three groups was not significantly different. The regression line exponents for RMR regressed on *M*_b_ did not match the expected (1.0) for geometric similarity (Table 1), but corresponded to the results reported in previous studies [15], [21], where RMR∝*M*_b_^0.67^ (Table 1). As RMR∝*M*_b_^0.67^ and UPL∝*M*_b_^0.33^, RMR should be proportional to UPL^2 (i.e., 0.67/0.33)^. The scaling exponent determined for RMR against UPL was significantly less than 2 for both diving and non-specialist groups (UPL^1.78 & 1.53^; Table 1). For the walkers, however, RMR did not change predictably with UPL (Table 1).

A GLM suggested that after controlling for the well-established relationship between size (*M_b_*) and RMR, and the group differences determined above, UPL is significantly positively correlated (coefficient = 6.77) with RMR (group: *F*_2,30_ = 5.238, *P* = 0.011; *M_b_*: *F*_1,30_ = 336.108, *P*<0.001; UPL: *F*_1,30_ = 43.481, *P*<0.001, model r^2^ = 0.954). Given the non-significant association between RMR and UPL for walking birds (RMA: Table 1), the GLM was repeated with non-specialist and diving species only. Again, locomotor group did not explain any variation in RMR (group: *F*_1,25_ = 0.429, *P* = 0.518; *M_b_*: *F*_1,25_ = 86.1441, *P*<0.001; UPL: *F*_1,25_ = 15.387, *P* = 0.001, model r^2^ = 0.973). Therefore, the group factor was removed from the GLM, leaving *M_b_* and UPL as covariates. As found for the GLM including all three groups, after accounting for the variation in RMR attributable to *M_b_*, the length of the UP was again significantly positively correlated (coefficient = 3.21) with RMR (*M_b_*: *F*_1,26_ = 88.035, *P*<0.001; UPL: *F*_1,26_ = 17.706, *P*<0.001, model r^2^ = 0.972).

**Figure 2:**
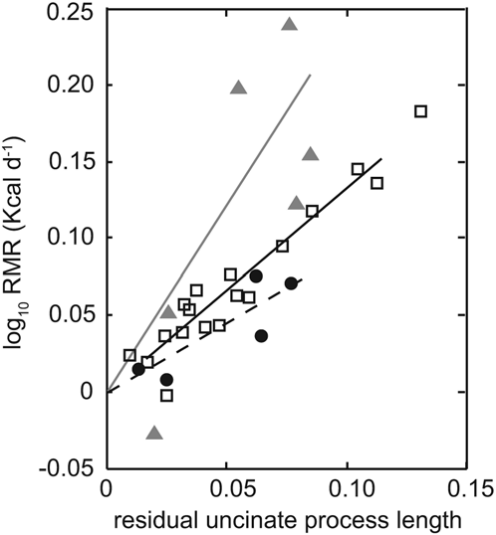
The relationship between RMR and residual uncinate process length (UPL). Plotted are the phylogenetically independent values derived using CAIC [32] and the phylogeny of Livezey and Zusi [31]. Diving birds (grey solid triangles and grey regression lines), non-specialists (open squares and black regression lines) and walkers (solid circles and dashed regression lines). The equations describing the lines of best fit were *y* = *x* ^2.43 (1.31−2.96)^ (*t* = 6.42, *n* = 6, *r* ^2^ = 0.85, *p*<0.001) for diving birds, *y* = *x* ^1.32 (1.21−1.42)^ (*t* = 26.69, *n* = 18, *r* ^2^ = 0.97, *p*<0.001) for non-specialists, and *y* = *x* ^0.91 (0.57−1.24)^ (*t* = 7.48, *n* = 5, *r* ^2^ = 0.91, *p*<0.005) for walkers respectively.

A similar result was found when using the RMR and residual uncinate process phylogenetically independent data. The length of the UP was positively correlated with RMR and this relationship varied between locomotor groups (Figure 2). Therefore, a longer uncinate process (when controlled for body mass) is found in birds with high RMRs relative to *M*_b_.

## Discussion

Our analysis indicates that the length of the uncinate process increases with body mass irrespective of the mode of locomotion used by the bird. UP are important structures for moving the ribs and sternum during ventilation. Sternal mass is primarily composed of the major flight muscles, the pectoralis and supracoracoideus. Geometric scaling of the major flight muscles to body mass has been calculated [35], although subsequent reports have suggested that pectoral mass scales with a slight negative allometry when looking at the non-passerines only [36]. An increase in rib length and sternal mass will necessitate a corresponding increase in process length [12]. Therefore, the increase in process length proportional to body mass is necessary as sternal mass increases. While process length scales isometrically with mass in all locomotor groups, UP are longest in diving birds, intermediate in non-specialists and shortest in walking species (Figure 1a; Table 1). The relationship between the locomotor mode of the bird and UPL was described by Tickle et al [12]. Elongated processes are likely to relate to the increased sternal length and low angle of the ribs to the backbone in diving birds [12]. These adaptations for a streamlined body facilitate efficient entry into, and locomotion in water and accordingly a greater mechanical advantage is required for respiratory movements of the relatively long sternum in divers. The relative increase in process length provides the necessary increase in effectiveness of the *Mm. appendicocostales* [12]. Contrastingly, walking species often have a relatively small sternal mass, relating to a reduction in flight muscle mass. This reduction coupled with a larger angle of the ribs to the backbone, means that a relatively small mechanical advantage may be sufficient for rib movements [12].

In this paper we have used the RMR of birds as a proxy for energy demand. For the birds used in this study, RMR scaled with *M*_b_ as expected and in line with previous predictions. While data for RMR is widely available in the literature, the validity of many reports has been questioned [21]. The lack of a standardised experimental protocol means that it is unclear how measures of BMR and/or RMR relate to each other. For example, metabolic rate is influenced by temperature and activity cycle [19], [20], variables which have not always been standardised between experiments. Although these are obvious limitations in the dataset, we consider RMR to be a valid measure of energy metabolism. However, considering that the form and function of the respiratory system may be adjusted to the demands of maximum exertion [37], maximal metabolic rate (MMR) may represent a more appropriate measure of metabolic demand. Unfortunately, the relative lack of MMR data prevents its use in comparative analyses like those presented in this study. However, the use of RMR as a proxy for MMR may be considered valid in the light of recent work in avian energetics. Comparative analyses have suggested a correlation between RMR and cold induced MMR [24], [38]. After removing the effect of body mass, RMR and MMR show similar scaling exponents [24]. It is unclear whether this relationship will hold for the wider range of species used in this study, since the Rezende et al, [24] analysis was dominated by data for passerines. The relationship does appear to hold ontogenetically for at least one species, however, the Australian Brush Turkey [39]. The relationship between RMR and flight MR, however, has not been extensively examined in birds, with the exception of some attempts to estimate the relationship [40], [41]. Therefore, while we must interpret our results carefully, it seems that RMR may be an adequate proxy for maximum metabolic demand.

A combination of the small RMR dataset that is available for walking species and the diversity within this group (a mixture of flightless and ground dwelling birds) may explain why there is no association between uncinate process length, body size, and RMR in these birds. The GLM analysis indicates that when we control for body size, increases in the length of the UP correspond to an increase in RMR. Therefore, birds with higher RMR for a given body mass have proportionately longer UP. Consequently, relatively longer UP appear to be associated with an elevated metabolic rate. Perhaps elongation of the UP facilitates an increase in metabolic demand by improving ventilation via the action of the UP in moving the ribs and sternum. Improving the work involved in ventilation may also explain the long UP found in diving species such as the penguins [42] and tufted ducks that have an elevated breathing frequency upon resurfacing after dives [43].

Of course, if it is advantageous, the question of why all birds don't have proportionally longer UP arises. Presumably, process elongation comes at a cost, possibly in terms of changing the shape of the associated musculature. Furthermore, UP act as levers for rib and therefore sternal movement and longer levers will not always provide a mechanical advantage depending on the overall body shape of the birds. Crucially, the rib cage, sternal morphology and associated musculature, appear to be driven by adaptations to different forms of locomotion.
